# Isolation and characterization of *Clostridium perfringens* strains isolated from ostriches (*Struthio camelus*) in Vietnam

**DOI:** 10.14202/vetworld.2020.1679-1684

**Published:** 2020-08-25

**Authors:** Tham Thi Nguyen, Hung Vu-Khac, Tan Duc Nguyen

**Affiliations:** 1 Department of Technology and Development Product, Institute of Veterinary Research and Development of Central Vietnam, Nha Trang City, Vietnam; 2 Department of Biotechnology, Institute of Veterinary Research and Development of Central Vietnam, Nha Trang City, Vietnam

**Keywords:** *Clostridium perfringens*, multiplex polymerase chain reaction, ostriches, toxin genes

## Abstract

**Background and Aim:**

*Clostridium perfringens* can cause enteritis in ostriches. The toxin release is believed to play a major role in determining pathogenesis properties of these pathogenic bacteria. This study was conducted to isolate and characterize *C. perfringens* strains from ostriches in Vietnam for identifying if particular virulence factors of these pathogenic bacteria are associated with enteritis progress in ostriches.

**Materials and Methods:**

The prevalence of *cpa, cpb, iA, etx, cpe*, and *cpb2* genes among *C. perfringens* isolates was determined by a multiplex polymerase chain reaction (PCR) method. The NetB toxin-encoding gene was detected by PCR and then sequenced to observe their variation. The expression of NetB toxin was checked by SDS-PAGE.

**Results:**

A total of 116 *C. perfringens* isolates were obtained from 318 fecal samples and 105 intestinal organs. Of 80 isolates from fecal samples, 33 isolates were from healthy and 47 isolates were from diseased ostriches. The results of multiplex PCR showed that all 116 *C. perfringens* strains from healthy and enteric disordered ostriches were positive for the alpha toxin-encoding gene (*cpa*). The cpe and *cpb2* genes were found in only one and five diseased ostriches, respectively. The *netB* gene was detected in 1/33 (3.03%) *C. perfringens* isolates from healthy ostriches, in 8/47 (17.05%) isolates from feces, and in 7/36 (19.44%) intestinal contents of diseased ostriches. The full-length sequences of 5 out of 15 *netB*-positive isolates from diseased ostriches showed 100% identity to each other as well as to the *netB* sequences available in GenBank. All of these five isolates produced NetB toxin *in vitro*.

**Conclusion:**

Type A is the most prevalent among *C. perfringens* isolates from ostriches in Vietnam. Especially, the study provides data emphasizing the role of NetB toxin in causing necrotic enteritis by *C. perfringens* in ostriches.

## Introduction

*Clostridium perfringens* can cause enteritis in ostrich chicks [1,2]. The bacterium is a normal inhabitant of the gut, and predisposing stress factors such as change of diet are thought to lead to its proliferation leading to the disease condition [2]. *C. perfringens* strains were previously classified into five specific toxinotypes (A-E) [3-5]. However, in a recent study, they have been proposed for seven toxinotypes (A-G) dependent on which major toxins (α - cpa, β - cpb, ε - etx, ι - iap, CPE - cpe, and NetB - netB) they produced [5,6]. The release of these toxins is believed to play a major role in determining pathogenesis properties of *C. perfringens* [4,7]. The role of other toxins produced by *C. perfringens*, such as enterotoxin and beta2 toxin, in causing enteritis is considered to be less significant [7,8], whereas the NetB toxin has been implicated as the major contributing factor to enteritis in chickens although other complementary factors may still exist [8-11].

In Vietnam, raising ostriches have been started since 1995, the ostrich breeding was imported from Zimbabwe and Australia [10]. According to data from General Statistics Office of Vietnam, in 2018, there were 56 provinces and cities situated in Vietnam involved in ostrich farming, providing 4660.1 million ostriches to slaughters. The symptoms of enteritis have been reported in ostrich farms in Vietnam; however, there is no report on the prevalence of *C. perfringens* in ostrich farms and hence no molecular characterization of this bird-derived isolates.

The present study investigated the prevalence of *C. perfringens* among ostriches raised in Vietnam. Toxinotypes and NetB production of the isolated *C. perfringens* were characterized to understand their potential pathogenic properties.

## Materials and Methods

### Ethical approval

The approval from the Institutional Animal Ethics Committee to carry out this study was not required as no invasive technique was used.

### Study period and location

The study was conducted from January 2016 to January 2019. We collected fecal and intestinal samples from ostrich farms located at Quang Binh, Quang Nam and Khanh Hoa provinces of Vietnam. The samples were transported on ice to the Department of Technology and Development Product, Institute of Veterinary Research and Development of Central Vietnam for testing.

### Sample collection and *C. perfringens* strains

A total of 318 fecal samples (158 from healthy and 160 from suspected necrotic enteritis [NE] ostriches which showed anorexia, ruffled feathers, depression, diarrhea, and listlessness) and 105 intestinal organs (small intestine, colon, and cecum) with typical lesions (thin intestinal wall, filled with gas, confluent mucosal necrosis of the small intestine, and depressed ulcers in the mucosal surface) were collected. The fecal samples were streaked onto blood agar plates containing 7% sheep blood agar and incubated in an anaerobic chamber at 37°C for 48 h. Colonies which showed dual hemolytic zones were picked and subcultured in Tryptose Sulfite Cycloserine agar (TSC agar, Oxoid, Merck) for purification and were grown in fluid thioglycollate (FTG, Merck) for toxin production. For the intestinal organs, samples were taken by scrubbing the intestinal inner wall of affected ostriches with cotton swabs then processed in the same way as fecal samples. The identity of the isolates was confirmed by their colonial and microscopical morphology, hemolytic pattern, Gram staining, and biochemical tests as previously described [12]. Each *C. perfringens* isolate from one ostrich was selected for further study.

### Determination of toxin-encoding genes of *C. perfringens* isolates

The multiplex polymerase chain reaction (PCR) reactions detecting *cpa*, *cpb*, *iA*, *etx*, *cpe*, and *cpb2* genes were performed as described by Songer and Bueschel [13]. The PCR reaction for *netB* detection was carried out as described by Keyburn *et al*. [10]. All primers applied in this study are listed in Table-1 [11,14,15].

**Table-1:** Primers applied in polymerase chain reactions detecting *Clostridium perfringens* toxin genes.

Toxin	Primer	Nucleotide sequence	Amplicon size (bp)	References
α-Toxin	Cpa-F	GCTAATGTTACTGCCGTTGA	324	[14]
	Cpa-R	CCTCTGATACATCGTGTAAG		
*β*-Toxin	Cpb-F	GCGAATATGCTGAATCATCTA	196	
	Cpb-R	GCAGGAACATTAGTATATCTTC		
*ε*-Toxin	etx-F	GCGGTGATATCCATCTATTC	655	
	etx-R	CCACTTACTTGTCCTACTAAC		
*ι*-Toxin	iA-F	ACTACTCTCAGACAAGACAG	446	
	iA-R	CTTTCCTTCTATTACTATACG		
*Cpe*	Cpe-F	GGAGATGGTTGGATATTAGG	233	
	Cpe-R	GGACCAGCAGTTGTAGATA		
Cpb2	Cpb2-F	AGATTTTAAATATGATCCTAACC	567	
	Cpb2-R	CAATACCCTTCACCAAATACTC		
*netB*	*netB-F*	GCTGGTGCTGGAATAAATGC	384	[11]
	*netB-R*	TCGCCATTGAGTAGTTTCCC		
*netB* (for sequencing)	netB(-100)F	CCAGTTATGTATAAATTTTGACCAGTT	1378	[15]
	netB(1278)R	AAACTTTAGTATTCCTCTCATTTTTTATCCC		

### Total DNA extraction

A single colony of each *C. perfringens* strain was suspended in 200 μL distilled water, boiled for 10 min, and then centrifuged at 10,000 × g for 10 min. The supernatants were collected and used as template DNA for PCR reactions.

### DNA sequencing of netB gene

The DNA samples were subjected to *netB* gene amplification reaction using netB(-100) F and netB(1278)R primers (Table-1) [14]. The PCR products were purified using QIAquick Gel Extraction Kit (QIAGEN – Germany) according to the manufacture’s instruction and sent to 1^st^ BASE – Singapore for sequencing.

### NetB toxin production and purification

*C. perfringens* isolates were cultured, supernatant harvested, and protein purified as described by Keyburn *et al*. [10]. The NetB protein (detected at approximately 33 kDa) was analyzed by SDS-PAGE. The protocol for SDS-PAGE was used as described in “A Guide to Polyacrylamide Gel Electrophoresis and Detection, BIO-RAD”.

### Results

### Isolation of *C. perfringens* infected in ostrich farms in Vietnam

A total of 116 *C. perfringens* isolates were obtained from 318 fecal samples and 105 intestinal organs. Of 80 isolates from fecal samples, 33 isolates were recovered from healthy ostriches and 47 isolates came from disordered birds. Only one isolate from each ostrich was considered. The results of biochemical tests proved that all bacterial isolates exhibited the characteristic features of *C. perfringens* as documented [12].

### Prevalence of toxin-encoding genes among *C. perfringens* isolates

**Figure-1:**
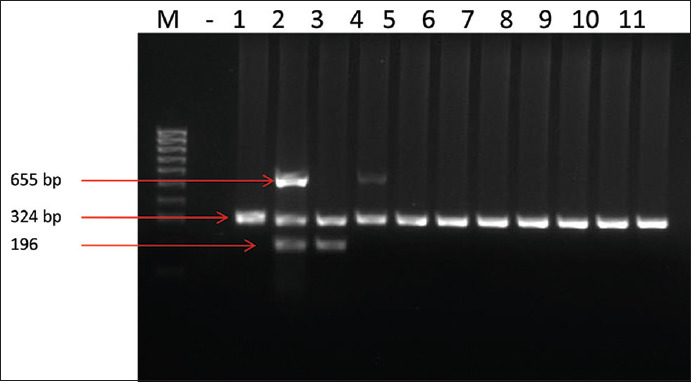
Multiplex polymerase chain reaction for toxins typing of *C. perfringens*. M: 100 bp DNA ladder maker, (−) negative control, lane 1: Positive control for type A, lane 2: Positive control for type B, lane 3: Positive control for type C, lane 4: Positive control for type D, lane 5-11: Samples.

**Figure-2:**
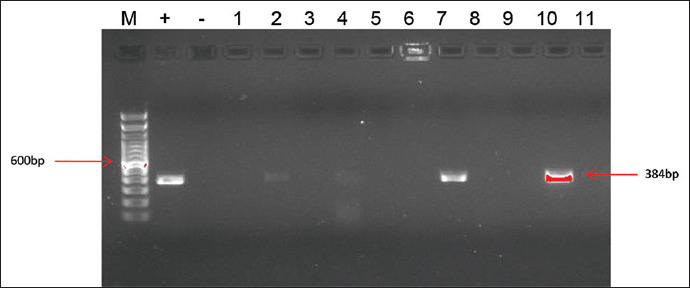
Polymerase chain reaction for netB gene detection. M: 100 bp DNA ladder, (+): Positive control, (−): Negative control, lane 1-11: Samples, lane 7, 10: Positive for netB gene.

The results of multiplex PCR showed that all *C. perfringens* strains regardless of sourced from healthy and enteric disordered ostriches were positive for the alpha-toxin gene (*cpa*) (Figure-1). The *cpe* gene which encodes *C. perfringens* enterotoxin was found in only one isolate from the diseased ostrich. As stated by the new typing scheme of Rood *et al*.[6], this strain belongs to type F as it carried both *cpa* and *cpe* genes. The gene encoding CPB2 toxin was detected in five diseased ostriches (three from fecal and two from intestinal samples). The PCR results of *netB* detection (Figure-2) recognized 16 strains positive for this gene. Except one strain carrying all three genes of *cpa*, *cpe*, and *netB*, 15 left strains were classified as type G following the Rood’s typing [6]. Our study also showed a clear difference of *netB* prevalence between the *C. perfringens* isolates from healthy and diseased birds. There were only 1/33 (3%) isolates from healthy ostriches detected for *netB* gene while 8/47 (17.05%) and 7/36 (19.44%) isolates, respectively, from fecal and intestinal samples of diseased birds positive for this gene.

### Analysis of netB sequences

We selected 5 out of 15 *netB*-positive isolates from diseased ostriches for *netB* amplification and sequencing. The analyzed sequences cover both the 969 bp coding sequence (CDS) region and 60 bp upstream promoter region including sequences potentially representing the RNA polymerase/sigma factor recognition sites (35: TTGAAA; 10: TATAAT) as well as the ribosomal binding site (AGGAGG). All the sequences derived from five isolates (GenBank accession number MT 032260-MT 032264) showed 100% identity to each other as well as to the *netB* sequences presently available in GenBank. No nucleotide variations in the CDS and promoter regions were observed. These results indicated that regardless of their geographic origin, *netB* gene is conserved among *C. perfringens*.

### Production of NetB toxin in *C. perfringens* isolates

**Figure-3:**
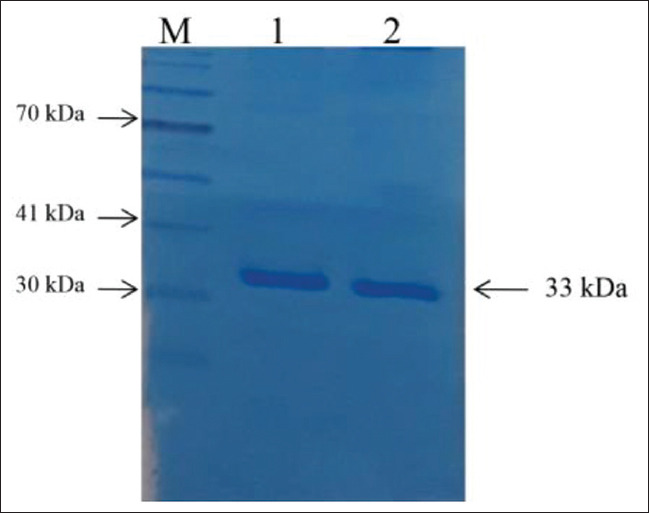
SDS-PAGE of purified NetB toxin stained with Coomassie blue. M: Pre-stained marker, Lanes 1-2: NetB-positive isolates from diseased ostriches.

Five *netB*-positive isolates from diseased ostriches and one isolate from healthy bird were selected for testing NetB toxin production *in vitro*. These strains were cultured for supernatant harvest, NetB toxin purified, and inspected on Coomassie blue-stained SDS-PAGE gel (Figure-3). All five isolates from diseased ostriches showed accumulated amount of NetB toxin in the supernatant with a molecular mass of approximately 33 kDa by SDS-PAGE analysis, whereas the *netB*-positive isolate from healthy bird did not produce this toxin.

## Discussion

This study is the first report of the prevalence and toxinotype characterization of *C. perfringens* isolates from ostriches raised in Vietnam. The strain identification was carried out by conventional morphological biochemical tests and toxin-encoding genes of *C. perfringens* isolates were detected by multiplex PCR reactions. The results of isolation showed that *C. perfringens* infection was found in 20.88% (33/158) healthy ostrich-derived fecal samples and 29.37% (47/160) of diseased birds. Especially, the infection percentage of *C. perfringens* in diseased ostrich intestine was determined to be higher (34.28%). The prevalence of *C. perfringens* among healthy ostriches in this study is lower than the result of Alimolaei and Ezatkhah study in Iran, where 46/118 (38,98%) samples were positive for *C. perfringens* [15]. However, this study showed a higher prevalence of *C. perfringens* in diseased ostriches compared to the result of Keokilwe *et al*. [16] in South Africa where *C. perfringens* was detected in only 20% (44/122) samples.

*C. perfringens* type A is the most common toxinotype in the environment and associated with food poisoning in humans [3]. In this study, we found all 116/116 (100%) *C. perfringens* strains carrying *cpa* gene. This finding was in agreement with the previous studies in Iran, where 30/30 *C. perfringens* isolates [17] and 46/46 *C. perfringens* isolates [15] were positive with *cpa* gene. In another study, Keokilwe *et al*. [16] also found that the *cpa* gene was the most prevalent (93,2%) among *C. perfringens* isolates from ostriches which showed enteritis symptoms in South African. Several studies have indicated that *cpe*-positive strains of *C. perfringens* from poultry occur in low number and can be less than 5% of global *C. perfringens* isolates [18-20]. These results are in agreement with our study as we found that only one *C. perfringens* isolate from ostrich was positive with *cpe* gene. Several studies have shown that the beta-2 toxin is not a critical factor in *C. perfringens* infection in particular cases of poultry [7,21] as its prevalence was documented to be low in diseased poultry but quite high in healthy ones [7]. In this study, we found only 5 (4,31%) isolates from diseased ostriches carried *cpb2* gene. It has been reported by Keyburn *et al*. [10] that NetB, a pore-forming toxin, is critical to NE development in chickens. They also reported that *netB* knockout mutants failed to produce NE in chickens, while such mutants complemented with the wild-type *netB* gene, caused NE. The present study revealed the prevalence of *netB*-positive *C. perfringens* isolates was 17.05% in fecal and 19.44% in intestinal samples of diseased ostriches, while only 3.03% strains from healthy ostriches carried *netB* genes (Table-2). Our results were in agreement with the investigation carried out in Iran [22], where 8/16 *C. perfringens* isolates from NE ostriches were positive for *netB* genes, while none of 20 isolates from healthy birds was positive for this gene. In another study, Keokilwe *et al*. showed that 7/43 *C. perfringens* isolates from 1-day to 3-month ostriches with enteritis symptoms were positive with *netB* genes [16]. The study of Abildgaard *et al*. has shown that NetB production was only observed in 4 out of 14 *netB*-positive *C. perfringens* isolates recovered from healthy chickens, whereas 12 out of 13 *netB*-positive isolates from NE chickens were determined to produce the NetB toxin [14]. In this study, we also found that all five *netB*-positive isolates from diseased ostriches accumulated NetB toxin *in vitro* as analyzed by SDS-PAGE, while the isolate from healthy ostrich did not produce this toxin.

**Table-2:** Prevalence of toxin-encoding genes in *Clostridium perfringens* isolates.

Samples	Number of isolates	Toxin genes of *Clostridium perfringens*							

		*Cpa*		*Cpe*		*Cpb2*		*NetB*	
			
		No. isolates	%	No. isolates	%	No. isolates	%	No. isolates	%
Fecal samples from healthy ostriches	33	33	100	0	0	0	0	1	3.03
Fecal samples from diseased ostriches	47	47	100	1	2.12	3	6.38	8	17.05
Intestinal organs from diseased ostriches	36	36	100	0	0	2	5.55	7	19.44
Total	116	116	100	1	0.77	5	4.31	16	13.79

Our results combined with these referenced ones support the suggestion of Keybum *et al*. [10] that NetB toxin plays an important role in causing enteritis disease in poultry. However, the present study indicated that there is a large population of *C. perfringens* isolates from diseased birds that do not carry *netB*. In agreement with this result, a study in Iran found that only 6.6% of the isolates from NE case of turkeys (*Meleagris gallopavo*) positive for *netB* and all isolates from flocks of mynah (*Acridotheres tristis*) and partridge (*Perdix perdix*) with severe NE symptoms negative for *netB* [23]. In another study, Ezatkhah *et al*. [24] also reported a low prevalence of *netB* gene (17.78%) in the isolates from broiler chickens clinically suspected to NE. According to these findings, NetB toxin may not be an obligate requirement for *C. perfringens* virulence or at least the presence of this toxin may not be essential for the disease process in all *C. perfringens* isolates. Another possible explanation to consider is that *netB*-negative isolates can cause disease in the field either by themselves or as part of a wider microbial consortium. Therefore, NE process with the roles of NetB toxin as well as alternative virulence factors and microflora in the association with *C. perfringens* needs further investigation.

For a better understanding of the role of NetB toxin role in *C. perfringens* pathogenesis, we sequenced five *netB* genes of five NetB toxin-producing isolates. The results showed that all of the analyzed sequences showed 100% identity to each other. No nucleotide variations in the CDS and the promoter regions were observed, indicating that *netB* gene is conserved among *C. perfringens* isolates in Vietnam. In the case of blasting only the 969 bp CDS region, we found 17 full-length *netB* gene sequences available in GenBank showing 100% identity to our sequences. However, there was only one sequence from *C. perfringens* isolate from Denmark (GU433324.1) [14] revealed 100% identity to our sequences that include both the CDS and promoter regions. We analyzed 20 *netB* gene sequences, including CDS and promoter regions available in GenBank to clarify how the *netB* genes of *C. perfringens* isolates in Vietnam are different from them. It is revealed that there is one A nucleotide at −58 bp position deleted in *netB* sequences of Vietnam isolates compared to other *netB* genes of *C. perfringens* isolates from other geographical regions in the world. The effect of this variation could be related to NetB expression regulation and need further studies.

## Conclusion

This study is the first report of the prevalence and toxinotype characterization of *C. perfringens* isolates from ostriches in Vietnam. The data presented in this study confirm that Type A is the most prevalent among *C. perfringens* isolates from poultry. In addition, the study provides data emphasizing the role of NetB toxin in causing necrotic enteritis in ostriches.

## Authors’ Contributions

TTN carried out the experiments and drafted the manuscript. HV and TDN conceived the original idea, supervised the project, and revised the manuscript. All authors have read and approved the final manuscript.

## Competing Interests

The authors declare that they have no competing interests.

## Publisher’s Note

Veterinary World remains neutral with regard to jurisdictional claims in published institutional affiliation.
